# New Developments in Exosomal lncRNAs in Cardiovascular Diseases

**DOI:** 10.3389/fcvm.2021.709169

**Published:** 2021-07-08

**Authors:** Zhu Yuan, Weiqiang Huang

**Affiliations:** Department of Geriatric Cardiology, Guangxi Key Laboratory Base of Precision Medicine in Cardio-Cerebrovascular Diseases Control and Prevention, Guangxi Clinical Research Center for Cardio-Cerebrovascular Diseases, The First Affiliated Hospital of Guangxi Medical University, Nanning, China

**Keywords:** exosomal lncRNA, cardiovascular disease, atherosclerosis, myocardial infarction, cardiac angiogenesis

## Abstract

Long non-coding RNAs (lncRNAs) are non-coding RNAs with lengths >200 nt and are involved in the occurrence and development of cardiovascular diseases (CVDs). Exosomes are secreted and produced by various cell types. Exosome contents include various ncRNAs, proteins and lipids. Exosomes are also important mediators of intercellular communication. The proportion of lncRNAs in exosomes is low, but increasing evidence suggests that exosomal lncRNAs play important roles in CVDs. We focused on research progress in exosomal lncRNAs in atherosclerosis, myocardial infarction, myocardial ischemia-reperfusion injury, cardiac angiogenesis, cardiac aging, rheumatic heart disease, and chronic kidney disease combined with CVD. The potential diagnostic and therapeutic effects of exosomal lncRNAs in CVDs are summarized based on preclinical studies involving animal and cell models and circulating exosomes in clinical patients. Finally, the challenges and possible prospects of exosomes and exosomal lncRNAs in clinical applications related to CVD are discussed.

## Introduction

Cardiovascular disease (CVD) is currently one of the main causes of death worldwide (1), and atherosclerosis and coronary heart disease are still representative challenges in CVD. In addition, the advent of an aging society has increased the cardiovascular burden, and the high incidence of rheumatic heart disease in developing countries and various concomitant cardiovascular diseases lead to final heart failure and other problems. The mainstream treatments, such as drugs, interventions, and surgery, cannot meet the needs of CVD patients with limited causes. In the case of myocardial infarction, emergency PCI or thrombolytic therapy can save the life of the patient but cannot block the adverse progression of the ischemic myocardium. For cancer patients, chemotherapeutic interventions can easily lead to premature heart failure (2). Therefore, exploring the molecular mechanisms underlying CVD and developing new treatments are critical for reducing CVD mortality.

LncRNAs are non-coding RNAs with lengths >200 nt. These factors were once considered noise in the genome. With the development of high-throughput sequencing, the continuous identification of functional lncRNAs has changed the view of junk RNA (3, 4). LncRNAs are found in various cells and circulation and play key roles in cardiovascular development and CVDs, and lncRNAs are expected to become targets for the treatment of CVDs (5). LncRNAs are transcribed through various regulatory methods, and their interaction with microRNAs (miRNAs) has become a hot spot for studying the functional mechanism of lncRNAs (6).

Exosomes are natural nanoscale cellular vesicles. The exosome surface is rich with specific marker proteins. After binding target cells, exosomes mainly introduce nucleic acids, proteins, and lipids through fusion with the plasma membrane and endocytosis. This content is delivered to the target cell for molecular regulation, representing an important mode of cell-to-cell communication (7). Although the level of lncRNAs in exosomes is low, their role cannot be ignored. In recent years, many studies investigating exosomal lncRNAs provided new insight into crosstalk in the tumor microenvironment (8, 9), and research in the field of CVDs has begun to emerge. This review focuses on the relationship between exosomal lncRNAs and CVD, including the production of exosomal lncRNAs and their mechanism in CVD. Current studies have shown that lncRNAs derived from exosomes are mainly involved in post-transcriptional regulation by sponge adsorption of miRNAs. We hope that this review can provide a basis for their future clinical application through current research investigating exosomes and exosomal lncRNAs in CVD.

## LncRNA Classification and Function

Similar to mRNAs, most lncRNAs are transcribed by RNA polymerase II (Pol II), capped at the 5′ end (m7G), tailed at the 3′ end (polyadenylation) and spliced, but lncRNAs lack an open reading frame and do not encode proteins for translation (10). According to the positional relationship between lncRNAs and protein-coding genes, lncRNAs can be divided into (1) sense lncRNAs that overlap with protein-coding genes; (2) antisense lncRNAs that are opposite to protein-coding genes; (3) lncRNAs from introns of protein-coding genes; (4) intergenic lncRNAs located between protein-coding genes; (5) lncRNAs derived from enhancers of protein-coding genes; (6) divergent lncRNAs transcribed from bidirectional promoters; and (7) circular RNAs (circRNAs) formed by the reverse splicing of protein-coding genes (11, 12). LncRNAs are located in the nucleus and cytoplasm and participate in the regulation of gene transcription through cis-acting or trans-acting mechanisms (10, 13–15). Before transcription, lncRNAs can bind proteins or RNAs for chromatin modification. For example, *LNCPRESS1* interacts with *SIRT6* to prevent the chromatin localization of *SIRT6*, maintain histone H3K56 and H3K9 acetylation, and activate transcription (10, 15, 16). During transcription, lncRNAs can mediate gene silencing by interfering with the recruitment of transcription factors and Pol II to prevent transcription (10, 15). For example, the transcription of the lncRNA *AIRN* can interfere with the recruitment of Pol ll and inhibit the transcription of *LGF2R* (17). LncRNAs can also form an R-loop triplet structure with DNA to complete the recruitment of transcriptional cofactors to the promoter, regulate chromatin accessibility, and promote transcription through lncRNAs transcribed by enhancers (10, 15). LncRNAs participate in regulation after transcription and translation. For example, lncRNAs bind miRNAs through a ceRNA mechanism that regulates target gene expression and interact with proteins to form lncRNA-protein complexes to regulate mRNA splicing and translation (10, 15).

## Exosome Biology

**Figure 1:**
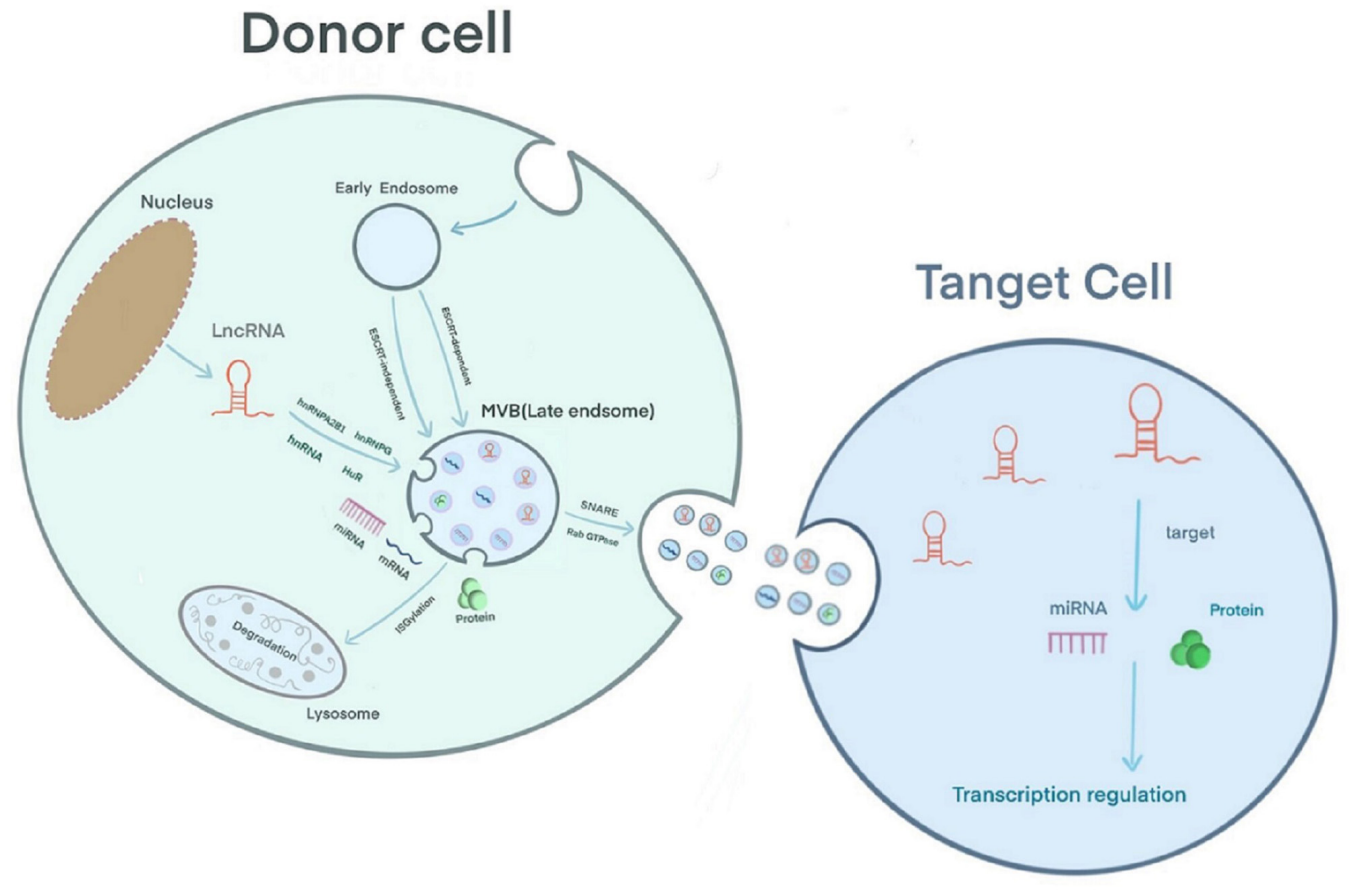
Exosome biology and the sorting and action mechanism of exosomal lncRNA.

Extracellular vesicles can be secreted by various cell types and are detected in body fluids. According to their biological mechanism and size, these vesicles are classified as exosomes, microcapsules, and apoptotic bodies. Exosomes are 30–150 nm in size; the plasma membrane initially invaginates to form early endosomes, and the endocytic membrane invades again to form late endosomes and multivesicular bodies (MVBs) (18). Studies have shown that there are two mechanisms underlying the formation of intraluminal vesicles (ILVs) in MVBs as follows: an ESCRT-dependent pathway and an ESCRT-independent pathway. The ESCRT complex includes ESCRT-0, ESCRT-I, ESCRT-II, ESCRT-III and the auxiliary proteins Alix and Vps4 (18, 19). ESCRT-0 recognizes ubiquitinated proteins outside the endosomal membrane. The complete recruitment of ESCRT-I and ESCRT-II occurs under the stimulation of phosphatidylinositol 3-phosphate (PIP3), hepatocyte growth factor-regulated tyrosine kinase substrate (HRS), ubiquitination of the cytosolic tails of endocytic proteins, or curved membrane topology. ESCRT-I binds the ubiquitinated cargo to the endosomal membrane and cooperates with ESCRT-II to open the endoluminal membrane for germination. ESCRT-III participates in the membrane rupture of the ILV neck under the recruitment of Alix, binds TSG101 and participates in the formation of the ESCRT-I complex. Under the action of a deubiquitinating enzyme, the ubiquitin mark is removed from the carrier protein to complete the sorting process. ESCRT-III is decomposed and recycled by the AAA-ATPase suppressor of potassium transport growth defect-1 (SKD1) for the next round of cargo recruitment (18–20). Studies have shown that ILVs can be formed in the absence of the ESCRT complex, indicating that the biological behavior of MVBs can be independent of the ESCRT pathway (21). For example, the lipid raft microdomains formed by ceramide and tetraspanin microdomains formed by CD63 trigger the transport of ILVs to MVBs (19, 20). Usually, MVBs are degraded by fusion with lysosomes after ISG-mediated modification of the TSG101 protein or are fused with the plasma membrane under the coordinated control of the cytoskeleton (microtubules and actin), soluble NSF attachment protein receptor (SNARE), and Rab GTPase to release exosomes (19) (Figure 1). A layer-by-layer analysis of the biological behavior of exosomes suggests that exosomes have great potential as a new type of biomarker for prediction and diagnosis in the field of cancer (22). Regarding CVD, exosomes have been reported to have potential application in the diagnosis and treatment of adriamycin-induced cardiotoxicity (23). In addition, Ghafarian et al. discussed the basic science and clinical application of exosomes in CVD (24).

## Exosomal lncRNA Sorting and Mechanism of Action

The mechanism by which lncRNAs are sorted into exosomes is still unclear. However, RNA-binding proteins (RBPs), including the hnRNP family [hnRNPA2B1 (25, 26), hnRNPG (27), hnRNPK (28)] and human antigen R (HuR) (29), have been confirmed to participate in the sorting of lncRNAs into exosomes (30). Furthermore, in sunitinib-resistant kidney cancer cells, hnRNPA2B1 has been shown to bind the 5′ end of *LNCARSR*, and knocking down the level of hnRNPA2B1 or mutating the binding site of hnRNPA2B1 reduces the level of *LNCARSR* in exosomes, suggesting that hnRNPA2B1 specifically facilitates *LNCARSR* loading into exosomes (31) (Figure 1). Previously, Zheng et al. introduced the mechanism of action of exosomal miRNAs, i.e., exosomal miRNAs affect the progression of CVD by binding target genes and mediating target mRNA silencing (32). The difference is that exosomal lncRNAs can be used for disease regulation through epigenetic modification. Furthermore, exosomal lncRNAs often act as sponges of endogenous miRNAs, mediating the expression of their target genes or activating related signaling pathways to produce biological effects. For example, Zang et al. found that the combination of the lncRNAs *UFC1* and *EZH2* resulted in H3K27 trimethylation and *PTEN* expression inhibition, which ultimately promoted the progression of non-small cell lung cancer (33). Zhuo et al. found that the exosomal lncRNA *FAM138B* derived from cancer cells can alleviate the progression of hepatocellular carcinoma by sponging *MIR-765* (34).

## Exosomal lncRNA in Cardiovascular Disease

In recent years, research investigating exosomal lncRNAs has become a trend. Especially in the field of cancer, several studies have shown that exosomal lncRNAs may become new biomarkers of and targets for cancer progression and treatment (35). In addition, similar studies investigated exosomal lncRNAs in various systemic diseases. For example, exosomal lncRNAs are expected to become a therapeutic target for lung diseases (36). In the field of CVD, Wang et al. found that the release of the exo-circRNA *HIPK3* from hypoxic cultured cardiomyocytes protects against cardiac microvascular endothelial oxidative damage by targeting the *MIR29A*/*IGF-1* pathway and promotes the proliferation and migration of cardiac endothelial cells through the *MIR29a*/*VEGF* axis (37, 38). Ni et al. discussed the relationship between exocrine-derived non-coding RNAs (lncRNAs and miRNAs) in endothelial cells and vascular smooth muscle cells and their mechanism in vascular senescence (39). Recently, Cheng et al. found *in vitro* that the exo-lncRNA *ZEB1-AS1* secreted by human umbilical vein endothelial cells induced by oxidized low-density lipoprotein (ox-LDL) enhances the damage caused by the *MIR-590-5P*/*ETS1* axis to human umbilical vein endothelial cells through the TGF-β/Smad pathway (40).

**Figure 2:**
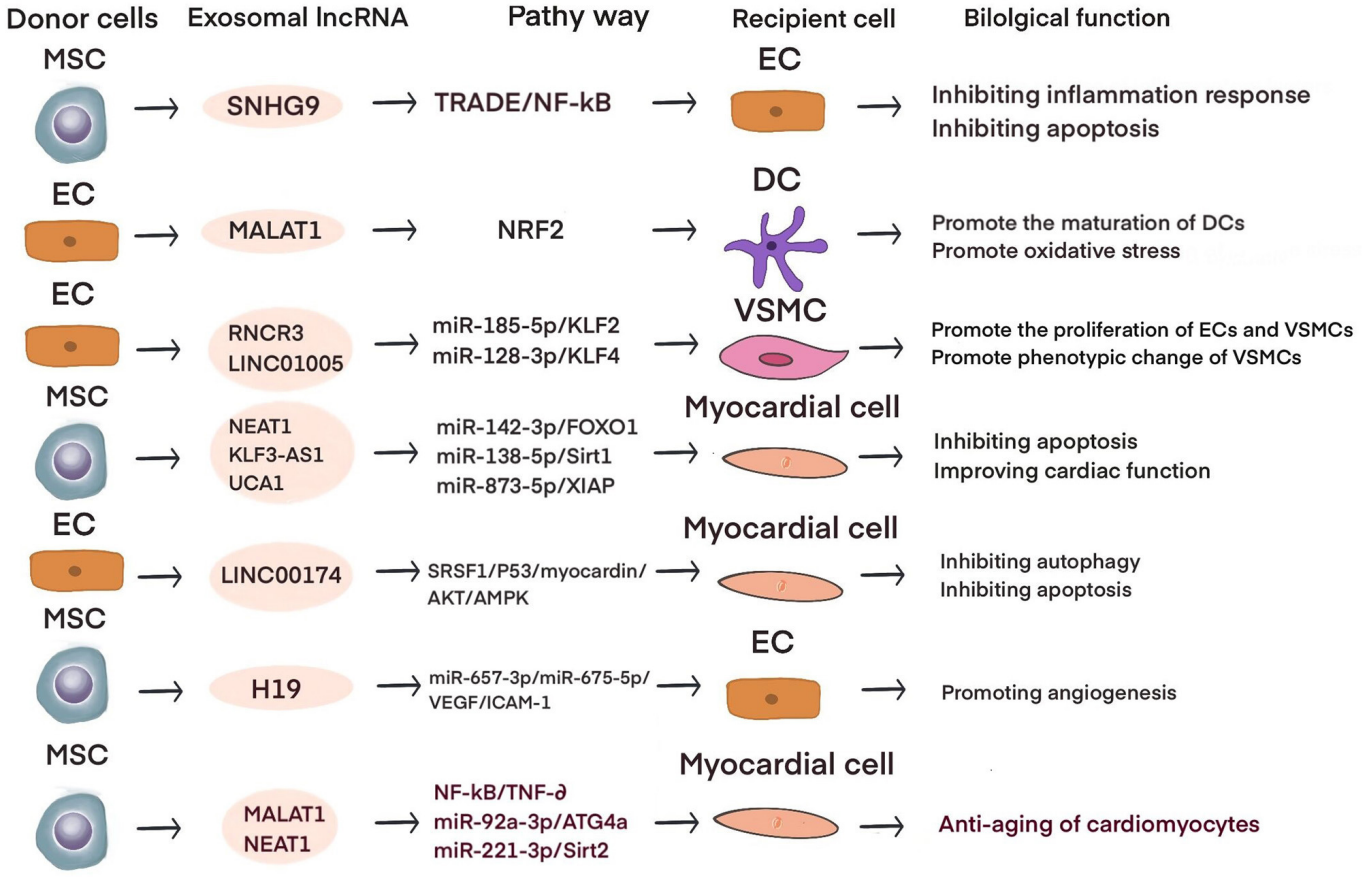
The role of exosomal lncRNA in preclinical studies of cardiovascular diseases.

**Figure 3:**
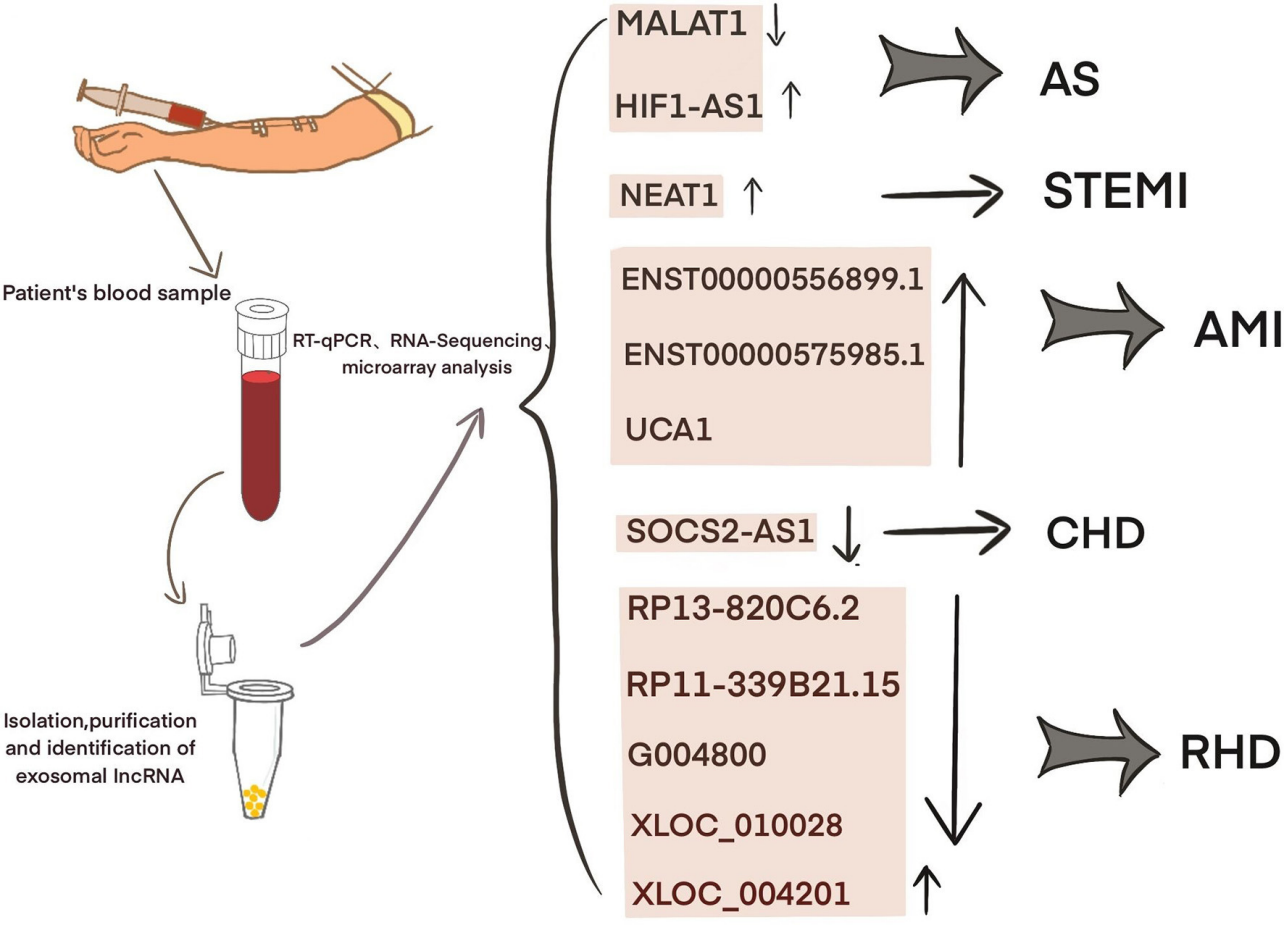
Exosomal lncRNA in the serum of CVD patients as potential biomarkers.

Current studies have shown that exosomal lncRNAs participate in the dynamic evolution of underlying cardiovascular diseases through various pathways, involving all aspects of their pathophysiology and potential treatment. For example, lncRNAs participate in the initial development of atherosclerosis, the occurrence of acute myocardial infarction (AMI) and ischemia-reperfusion injury, cardiac angiogenesis, repair and protection against cardiac aging (Table 1 and Figure 2). The differential changes in lncRNAs in patient plasma exosomes make them potential biomarkers for the diagnosis and treatment of CVDs (Table 2 and Figure 3).

**Table 1:** Progress of exosomal lncRNA in preclinical study of CVD.

**LncRNA**	**Donor cell**	**Target cell**	**Pathway**	**Type of CVD**	**Function**	**References**
RNCR3	HUVEC	VSMC	miR-185-5P/KLF2	AS	Promote the proliferation of ECs and VCMCs; inhibition of ECs apoptosis	(41)
MALAT1	HUVEC	iDC	NRF2	AS	Promote the maturation of DCs; promote the accumulation of oxidative stress	(42)
	HUVEC	THP-1	-	AS	Promote the polarization of M2 macrophages	(43)
	hCVPC	Cardiomyocyte; HUVEC	MiR-497	AMI	Inhibit cardiomyocyte apoptosis; promote angiogenesis of ECs	(44)
	Cardiomyocyte		miR-92a/KLF2/CD31	AMI	Reduce the area of myocardial infarction;Improve cardiac function; promote angiogenesis after myocardial infarction	(45)
	UMSC	H9C2	NF-κB/TNF-α	Cardiac aging	Delay cardiac aging	(46)
	AD-MSC	Cardiomyocyte	miR-92a-3p/ATG4a	Cardiac aging	Improve cardiac mitochondrial metabolism ;delay the senescence of cardiac myocytes	(47)
GAS5	THP-1	HUVEC	-	AS	Promote apoptosis of vascular endothelial cells	(48)
SNHG9	ADSC	HUVEC	TRADD/ NF-κB	AS	Inhibit the release of inflammatory factors ;inhibition of endothelial cell apoptosis	(49)
LINC01005	HUVEC	VSMC	miR-128-3P/KLF4	AS	Promote phenotypic transformation, proliferation and migration of vascular smooth muscle	(50)
NEAT1	ADMSC	HiPSC-derived cardiomyocytes	miR-142-3p/ FOXO1	MI	Anti-oxidative stress ;inhibit cardiomyocyte apoptosis	(51)
	BM-MSC	Cardiomyocyte	miR-221-3p/Sirt2	Cardiac aging	Anti-aging of cardiomyocytes	(52)
AK139128	cardiomyocyte	CF	-	MI	Inhibit the proliferation of cardiac fibroblasts and promote apoptosis	(53)
KLF3-AS1	hMSC	H9c2	miR-138-5p/ Sirt1	MI	Inhibit the release of inflammatory factors; attenuate pyroptosis of cardiomyocytes	(54)
ENSRNOT00000039868	PMN	Cardiomyocyte	PDGFD/AKT	MIRI	Anti-oxidative stress of cardiomyocytes;inhibit cardiomyocyte apoptosis;reduce I/R injury	(55)
UCA1	hUCMSC	CMEC	miR-143/Bcl-2/Beclin1	MIRI	Inhibition of cardiomyocyte autophagy;inhibition of myocardial apoptosis;reduce H/R injury	(56)
	hMSC	H9C2	miR-873-5p/XIAP	MI	Inhibit cardiomyocyte apoptosis;improve cardiac function	(57)
LINC00174	EC	cardiomyocyte	SRSF1/ P53/ myocardin/AKT/AMPK	MIRI	Inhibition of autophagy and apoptosis of cardiomyocytes	(58)
H19	MSC	H9C2; HUVEC	miR-675-3p/miR-675-5p/VEGF/ICAM-1	MI	Promote angiogenesis of ECs ;inhibit cardiomyocyte apoptosis ;inhibit myocardial fibrosis; improve cardiac function	(59)
ZFAS1	human cardiomyocyte	CF	miR-4711-5p/WNT/β	Myocardial fibrosis	induce myocardial fibrosis	(60)

**Table 2:** The role of serum exosomal lncRNA in cardiovascular disease.

**Exosomal lncRNA**	**Expression**	**Disease**	**Function**	**References**
HIF1a-AS1	Up	AS	Diagnostic	(61)
NEAT1	Up	STEMI	Diagnostic	(62)
ENST00000556899.1; ENST00000575985.1	Up	AMI	Prognostic	(63)
UCA1	Up	AMI	Diagnostic	(57)
SOCS2-AS1	Down	CAD	Diagnostic	(64)
RP13-820C6.2 RP11-339B21.15 G004800 XLOC_010028	Down	RHD	Diagnostic; Therapeutic	(65)
XLOC_004201	Up			

## Atherosclerosis

Atherosclerosis (AS) is the most common type of CVD and the root cause of myocardial infarction. The main risk factors are aging, hyperlipidemia, obesity, hypertension, and smoking. Endothelial injury is considered the first step in the development of atherosclerosis. Subsequently, after inflammatory cell infiltration, vascular smooth muscle cells (VSMCs) proliferate and activate, exacerbating plaque formation. Therefore, targeting endothelial dysfunction, inflammatory cells and the phenotypic transition of VSMCs are critical for the study of the development and treatment of atherosclerosis. For example, exo-*MIR143*/*MIR145* secreted by endothelial cells expressing *KLF2* can communicate with smooth muscle cells, increase the expression of dedifferentiation-related genes, and reduce atherosclerosis in ApoE-/- mice (66). Macrophage-derived exo-*MIR99A*/*MIR146B*/*MIR378A* targets NF-κB/TNF-α signaling to inhibit inflammation, promote macrophage M2 polarization, and reduce the area of atherosclerotic necrosis (67).

Obesity is a risk factor for atherosclerosis; obesity promotes endothelial cell (EC) dysfunction, accelerates endothelial cell apoptosis and the release of inflammation, and promotes the development of atherosclerosis (68, 69). Exosomal lncRNAs are involved in endothelial dysfunction. For example, lncRNA *SNHG9* overexpression in adipose stem cell-derived exosomes can inhibit endothelial cell apoptosis, inhibit NF-κB signaling, and reduce inflammation. *TRADD*, which is the target gene of *SNHG9*, is negatively associated with *SNHG9* and inversely regulates the protective effects of *SNHG9* to inhibit EC disorders (49).

M2 macrophages can fight the progression of atherosclerosis (67). Exosomal lncRNAs mediate communication between ECs and immune cells. For example, Huang et al. found that the expression level of the lncRNA *MALAT1* was significantly increased in exosomes secreted by ox-LDL-stimulated human umbilical vein endothelial cells (HUVECs) and that coculture with these exosomes increased THP-1 cell expression of *MALAT1* and promoted the polarization of M2 macrophages (43). A study by Chen et al. showed that the expression of the lncRNA *GAS5* in exosomes derived from THP-1 cells under ox-LDL stimulation was significantly upregulated. Exosomes secreted by ox-LDL-stimulated THP-1 cells with *GAS5* knockout were cocultured with HUVECs, and PHK67-labeled exosomes were internalized by HUVECs and significantly reduced HUVEC apoptosis. In contrast, exosomes secreted by ox-LDL-stimulated *GAS5*-overexpressing THP-1 cells could accelerate HUVEC apoptosis (48). Hong et al. found that the expression of *MALAT1* was significantly reduced in the serum of patients with atherosclerosis and exosomes derived from ox-LDL-stimulated HUVECs. Exosomes derived from ox-LDL-stimulated HUVECs overexpressing *MALAT1* are taken up by dendritic cells (DCs) and can inhibit DC maturation, activate NRF2 signaling, and inhibit reactive oxygen species (ROS) accumulation in DCs. Silencing *MALAT1* results in the opposite effects. Similarly, in a mouse model of atherosclerosis, ox-LDL-stimulated exosomes exacerbated atherosclerosis in mice. This finding shows that the expression of *MALAT1* is decreased in vascular endothelial cell-derived exosomes induced by ox-LDL, inhibits NRF2 signaling, leads to ROS accumulation and DC maturation, and accelerates the development of atherosclerosis (42).

Exosomal lncRNAs mediate the interaction between endothelial cells and VSMCs. For example, Shan et al. found that apoE-/- mice expressed increased levels of the lncRNA *RNCR3* during the atherosclerotic stage, and RNA fluorescence *in situ* hybridization revealed RNCR3 localization in endothelial cells and arterial smooth muscle cells. After knocking out *RNCR3 in vivo*, the endothelial cell coverage and smooth muscle proliferation were significantly reduced. *In vitro* cell experiments showed that after knocking out *RNCR3*, HUVEC proliferation slowed, and apoptosis accelerated. After coculturing HUVEC-derived exosomes (HUVEC-exos) with VSMCs, VSMC proliferation and migration were inhibited. Biological analyses and luciferase assays show that HUVEC-exo-*RNCR3* targets the *MIR-185-5P*/*KLF2* axis to promote the proliferation of ECs and VSMCs and plays a protective role in the development of atherosclerosis (41). Zhang found that coculturing ox-LDL-stimulated HUVECs-exo-*LINC01005* with VSMCs significantly downregulated the smooth muscle cell contraction markers α-SMA and SM22a and upregulated the VSMC proliferation marker OPN, indicating that HUVECs-exo-*LINC01005* promoted phenotypic changes in VSMCs. The database and luciferase reporter assay show that HUVECs-exo-*LNC01005* mediates phenotypic changes in endothelial cells and VSMC proliferation through the *MIR-128-3P*/*KLF4* axis (50). It is suggested that the communication of exosomal lncRNAs among ECs, VSMCs and immune cells is mainly involved in the progression of atherosclerosis by targeting miRNAs or directly regulating the expression of genes.

The differences in the expression of exosomal lncRNAs in peripheral circulation in patients with atherosclerosis may represent a potential biomarker for the diagnosis of atherosclerosis. For example, Song et al. found significantly low expression of exosomal *SNHG9* in the plasma of obese patients and revealed that this level was associated with endothelial dysfunction (49). Hong et al. found that a low expression of exosomal *MALAT1* in the serum of patients with atherosclerosis led to DC maturation and promoted the progression of atherosclerosis (42). Wang et al. examined 65 blood samples from atherosclerosis patients and 68 blood samples from healthy volunteers and found that the blood exosomal concentration and exo-lncRNA *HIF1A-AS1* expression in the atherosclerosis patients were significantly higher than those in the healthy group. A receiver operating characteristic (ROC) curve analysis showed that the areas under the curve of the plasma exosomes and exo-*HIF1A-AS1* were 0.856 and 0.823, respectively. This finding indicates that plasma exo-*HIF1A-AS1* may be a potential biomarker for the diagnosis of atherosclerosis (61). It is suggested that the differential expression of serum exosomal lncRNAs in patients with atherosclerosis may play an auxiliary role in diagnosis.

## Myocardial Infarction

Myocardial infarction is caused by coronary artery stenosis or occlusion, and the myocardium is in a state of ischemia and hypoxia. Apoptosis and fibrosis of ischemic and hypoxic cardiomyocytes lead to ventricular remodeling, which, in turn, causes arrhythmia and heart failure. Since the 21st century, the performance of cell therapy in the treatment of myocardial regeneration has been unsatisfactory. The paracrine mechanism that plays a major role in cell therapy has given high hopes regarding the use of exosomes characterized by non-cellular therapy (70–74). For example, in a mouse model of myocardial infarction, exosomes derived from mouse embryonic stem cells promote survival and proliferation in cardiac progenitor cells by delivering miR-294 (75). Intramuscular injection of exosomes derived from cardiosphere-derived cells can reduce acute and chronic myocardial infarction injury in pigs, reduce the area of myocardial infarction, reduce the area of scars, and combat cardiac remodeling (76).

In addition to exosomal miRNAs, mesenchymal stem cell (MSC)-derived exosomal lncRNAs also play important roles in myocardial infarction. For example, Che et al. found that the exosomal lncRNA *NEAT1* secreted by migration inhibitory factor (MIF)-induced human adipose mesenchymal stem cells (ADMSCs) inhibits H2O2-induced cardiomyocyte apoptosis. The database and dual luciferase gene reports show that the ADMSC^MIF^-derived exosomal *NEAT1* reduces H2O2-induced oxidative stress in cardiomyocytes and reduces cardiomyocyte apoptosis through the *MIR-142-3p*/*FOXO1* axis (51). Mao et al. detected the lncRNA *KLF3-AS1* in exosomes secreted by human MSCs (hMSCs). Injection of hMSC-exos in a rat myocardial infarction model significantly reduced the expression of the proinflammatory factors IL-1β and IL-18 in cardiomyocytes and the expression of NLRP3, Asc, and Caspase-1, indicating that hMSC-exos induced cardiomyocyte pyrolysis. In an *in vitro* hypoxic cardiomyocyte model, the protective effect of hMSC-exos was consistent with that observed in the *in vivo* experiments. Database and dual luciferase gene reporter experiments showed that the hMSC-exo-*KLF3-AS1*/*MIR-138-5p*/*SIRT1* axis mediates protection in hypoxic cardiomyocytes (54). Sun et al. performed cell and animal experiments and showed that the exosomal lncRNA *UCA1* secreted by hypoxia-induced human bone marrow MSCs (BMSCs) sponges *MIR-873-5p*, regulates *XIAP*, and enhances AMPK phosphorylation in cardiomyocytes. This lncRNA also increases the expression of antiapoptotic proteins, such as p53, bax, and cleaved caspase-3, and inhibits the expression of the apoptotic protein bcl-2 (57).

Studies have shown that exosomal lncRNAs mediate communication between cardiomyocytes and cardiac fibroblasts (CFs) in the context of myocardial infarction. For example, Wang et al. found that coculturing hypoxic cardiomyocyte-derived exosomes and CFs significantly inhibited CF proliferation, migration and invasion and promoted apoptosis. An RNA-seq analysis revealed the differential expression of the lncRNA AK139128. *In vivo* experiments showed that exo-AK139128 exacerbated CF apoptosis in rats with myocardial infarction. Western blot analyses showed that the expression of the apoptotic protein bcl-2 increased, while the expression of the antiapoptotic protein bax decreased. This finding shows that exosomal AK139128 secreted by hypoxic cardiomyocytes promotes CF apoptosis (53). The above studies show that in addition to exosomal lncRNAs derived from mesenchymal stem cells, which are expected to become therapeutic targets for the treatment of infarcted myocardium, exosomal lncRNAs between cardiac cells also have potential therapeutic significance and research value.

Recent studies have shown that significant differences exist in plasma exosomal lncRNAs in patients with myocardial infarction, suggesting that these factors may have an auxiliary diagnostic effect. For example, Chen et al. found that the serum expression of exosomal *NEAT1* and *MMP9* in patients with ST-segment elevation myocardial infarction (STEMI) was significantly higher than that in patients with unstable angina and non-myocardial infarction, while the level of *MIR204* was relatively lower. Spearman tested the correlation among the three factors and found that *NEAT1* was positively correlated with the *MMP9* levels, but *NEAT1* was not significantly correlated with *MIR204, MIR204*, or *MMP9*. A logistic regression analysis revealed that *NEAT1, MIR204*, and *MMP9* are independent predictors of STEMI (62). Zheng et al. found that ENST00000556899.1 and ENST00000575985.1 were significantly upregulated in circulating exosomes from AMI patients and control patients. An ROC analysis showed that ENST00000556899.1 and ENST00000575985.1 have areas under the myocardial infarction curve of 0.661 and 0.751, respectively, and are positively correlated with inflammatory biomarkers, myocardial infarction prognostic indicators, and myocardial injury markers (63). Sun et al. showed that the plasma expression of the exosomal lncRNA *UCA1* in patients with myocardial infarction was increased. An ROC analysis showed that human plasma exosomal *UCA1* may be a non-invasive biomarker for the diagnosis of AMI (57). Liang et al. enrolled 227 subjects and divided them into a coronary heart disease group, an early coronary heart disease group, and a normal coronary artery control group. Gene chip detection and RT-PCR showed that the exo-lncRNA *SOCS2-AS1* was significantly downregulated in the coronary heart disease group. A Pearson correlation analysis showed that the plasma exo-*SOCS2-AS1* levels were negatively correlated with the LPA and PLT levels. Univariate and multivariate logistic regression analyses showed that the plasma exosomal SOCS2-AS1 levels were an independent protective factor for coronary heart disease (64). These data suggest that exosomal lncRNAs may play an auxiliary role in the prediction and diagnosis of patients with myocardial infarction.

## Myocardial Ischemia-Reperfusion Injury

Myocardial ischemia-reperfusion injury (MIRI) refers to the pathological process during which the ischemic myocardium returns to normal perfusion after a coronary artery is partially or completely occluded, which, in turn, exacerbates myocardial injury. In clinical practice, MIRI is common in patients with myocardial infarction after emergency percutaneous coronary intervention (PCI) or thrombolytic therapy (77). The potential molecular mechanisms involved include oxidative stress, intracellular calcium ion overload, rapid recovery of PH, opening of the mitochondrial permeability transition pore (MPTP), the inflammatory response, and late myocardial reperfusion injury (77, 78). In recent years, treatments for MIRI have included ischemic preconditioning (IPC), ischemic post-processing (IPost), remote ischemic conditioning (IPC), the use of drugs to prevent myocardial reperfusion injury, therapeutic hyperbaric oxygen (HBO) and hypothermia (77–79). Recent studies have shown that the cardioprotective effect of IPC is mediated by exosomes (80). For example, exosomes derived from rat fibroblasts can protect cardiomyocytes after ischemia by targeting *MIR-423-3p*/*RAP2C* to reduce MIRI (81).

Similarly, exosomal *UCA1* derived from hMSCs was discovered by Sun et al. to exert myocardial protection after myocardial infarction (57). Diao et al. found that exosomes derived from human umbilical cord blood MSCs (hUCMSCs) could inhibit hypoxia/reoxygenation (H/R)-induced cardiac microvascular endothelial cell (CMEC) apoptosis in Sprague-Dawley rats. In addition, hUCMSC-exos can inhibit macrophage autophagy under hypoxia. In an *in vivo* IR rat model, injection of hUCMSC-exos improved the ultrastructure of rat CMECs, inhibited apoptosis, and reduced the vascular endothelial injury markers TM and vWF. An RT-qPCR analysis verified that hUCMSC-exos were enriched with *UCA1*. In addition, dual luciferase reporter and RNA pull-down experiments showed that hUCMSC-exo-*UCA1* inhibited autophagy and apoptosis in H/R-injured rat CMECs by regulating the *miR143*/*BCL-2*/*BECLIN1* axis (56).

Recent studies have shown that exosomal lncRNAs are involved in the communication between polymorphonuclear cells (PMNs) and IR cardiomyocytes. For example, Zhai et al. used an H/R cardiomyocyte model and showed that PMN-exos stimulated by the calcium sensitive receptor (CaSR) activator cinacalcet were taken up by cardiomyocytes, increased the expression of p-AKT and Bcl-xL, reduced the production of NOX2 and ROS, and reduced cardiomyocyte apoptosis, while the AKT inhibitor HY15186 could reverse these effects. A high-throughput sequencing analysis of CaSR-PMN-exos found differential expression of the lncRNA ENSRNOT00000039868 (lncRNA 39868) and predicted PDGFD as the target protein of lncRNA 39868. siRNA-mediated silencing of the lncRNA 39868 significantly reduced PDGFD mRNA expression. *In vivo* experiments showed that after intravenous injection of CaSR-PMN-exos via the tail vein in rats, the area of myocardial infarction and MDA secretion were significantly reduced, the SOD levels were increased, and cardiac function was improved. These results suggest that CaSR-PMN-exosomal lncRNA 39868 upregulates the protein PDGFD, regulates these effects through the AKT pathway, and reduces MIRI (55).

Exosomal lncRNAs derived from endothelial cells have protective effects on IR cardiomyocytes. For example, Su et al. measured the expression of exosomal *LINC00174* derived from vascular endothelial cells. After coculturing H/R cardiomyocytes with exo-*LINC00174 in vitro, P53* was inhibited, and P53-induced autophagy enhancement and cardiomyocyte apoptosis were inhibited. In contrast, the expression of *SRSF1* was increased. RNA pull-down experiments and RIP verified the binding of *LINC00174* to *SRSF1*. In I/R mice, AKT/AMPK phosphorylation was increased, indicating that myocardial autophagy was activated, but *P53* knockout inhibited autophagy activation and cardiomyocyte apoptosis. A dual luciferase reporter assay confirmed that *MYOCARDIN* was the binding site of *P53*. Vascular endothelial cell exo-*LNC00174* bound *SRSF1* to inhibit *P53* expression, *MYOCARDIN* transcription, AKT/AMPK activation, and ultimately autophagy activation and apoptosis in I/R cardiomyocytes (58). These results suggest that the mechanism of cardiomyocyte protection mediated by exosomal lncRNAs in myocardial ischemia-reperfusion injury involves crosstalk among mesenchymal stem cells, polymorphonuclear cells and endothelial cells. The study of exosomal lncRNAs in myocardial ischemia-reperfusion still needs more exploration.

## Cardiac Angiogenesis

Currently, revascularization treatment for AMI or ischemic cardiomyopathy mainly involves PCI, coronary artery bypass grafting and thrombolysis to restore the myocardial blood supply and save the dying myocardium. However, MIRI follows, and for patients who cannot undergo surgery, there is an urgent need for a new alternative therapy. In addition to cell therapy to rescue and restore cardiomyocytes, increasing research suggests that promoting the survival of cardiac endothelial cells, treating cardiac angiogenesis, and mediating the recanalization of cardiac collaterals have great therapeutic potential for the treatment of myocardial infarction. Examples include angiogenesis gene therapy (82), proangiogenic stem cell therapy (83), angiogenic ncRNA therapy (84), and exosome- and exosome-derived proangiogenic ncRNA therapy (85–88). For example, a study by Ma et al. showed that exosomes derived from murine BMSCs can deliver *MIR132*, induce tube formation in HUVECs *in vitro*, and promote angiogenesis in the ischemic area of the infarcted heart in mice (89).

Exosomal lncRNAs derived from stem cells can promote endothelial cell proliferation and angiogenesis after myocardial infarction. For example, Q. Wu et al. found that hypoxia-induced cardiovascular progenitor cells derived from human pluripotent stem cell (hCVPC)-EVs could improve heart function in mice with myocardial infarction, and immunohistochemistry studies showed that *CD31* and α-SMA expression increased. Similarly, *in vitro* experiments showed that hCVPC-EVs can reduce the damage induced by oxygen glucose deprivation (OGD) to cardiomyocytes and promote endothelial cell migration and tube formation. RNA sequencing and RT-qPCR analyses showed that the expression of *MALAT1* in hypoxic hCVPC-EVs was significantly increased, and knocking down the expression of *MALAT1* inhibited angiogenesis. Database and dual luciferase gene reporter assays suggest that *MALAT1* targets *MIR497* (44). After culturing atorvastatin-treated (ATV)-MSC-exos with HUVECs *in vitro*, Huang et al. showed that HUVECs treated with ATV-MSC-exos had enhanced tube-forming abilities. When cocultured with H9C2 cardiomyocytes stimulated with hypoxia and serum deprivation (H/SD), HUVEC-exos increased the survival rates of cardiomyocytes under H/SD conditions and inhibited myocardial fibrosis. In addition, rats with myocardial infarction were injected with ATV-MSC-exos, resulting in improved heart function, inhibited cardiomyocyte apoptosis, reduced inflammatory cell infiltration, and increased blood vessel density. The sequencing of ATV-MSC-exos demonstrated that the lncRNA *H19* was highly expressed, and the protective effect of ATV-MSC-exos on cardiomyocytes and the promotion of endothelial cell angiogenesis were eliminated after *H19* knockout. Furthermore, *MIR-675-3p, MIR-675-5p, VEGF*, and *ICAM1* were positively expressed with *H19*. These results suggest that atorvastatin increases the secretion of *H19* exosomes by rat mesenchymal cells, activates *VEGF* and *ICAM1* by regulating the expression of *MIR*−*675-3p* and *MIR*−*675-5p*, and promotes angiogenesis in the endothelium after myocardial infarction (59).

Studies have shown that HBO can promote the expression of angiogenic exosomal lncRNAs and mediate angiogenesis after myocardial infarction. For example, Shyu et al. found that HBO-induced cardiomyocyte exosomes increased the expression of *MALAT1*, decreased the expression of *MIR92A*, increased the expression of *KLF2* and *CD31*, reduced the area of myocardial infarction and inhibited heart remodeling. A luciferase activity assay verified that the target gene of *MIR92A* was *KLF2*. In an *in vitro* rat cardiomyocyte hypoxia model, an HBO intervention promoted the expression of exosomal *MALAT1* more than hypoxia stimulation. HBO induces the enrichment of exo-*MALAT1* derived from cardiomyocytes, regulates the expression of *CD31* and *KLF2* through the MIR92A/KLF2 axis, and promotes cardiac angiogenesis after myocardial infarction (45). The metastasis of angiogenic lncRNAs in exosomes is of great significance for ischemic diseases. There have been many reports in the field of cancer. We look forward to more studies concerning angiogenesis in exosomal lncRNAs. It is believed that exosomal lncRNAs could be a new target for the treatment of ischemic cardiomyopathy.

## Cardiac Aging

Cellular aging is an irreversible factor in the occurrence and development of CVD. Aging leads to excessive oxidative stress, chronic low-grade inflammation, telomere shortening, autophagy, and mitochondrial dysfunction (90–92). Aging also mediates communication between cardiomyocytes and endothelial cells, fibroblasts, and immune cells (93) and promotes vascular wall endothelial damage, atherosclerosis, myocardial fibrosis, coronary heart disease, and heart failure (94, 95). Currently, the strategies applied to delay cardiac aging include repairing mitochondrial dysfunction (96), targeting cardiac stem cell senescence and senescence-associated secretory phenotype changes (97), inducing autophagy (90), and hydrogen sulfide-mediated regulation of senescence signals (98). It has been reported that extracellular vesicles mediate cell senescence (99). Recently, Lei et al. found that the transfer of proliferating cell nuclear antigen from MSC-EVs derived from neonatal umbilical cords could reverse aging in adult BMSCs. In mice, UCMSC-EVs could delay the aging phenotype in aging mice and reduce degeneration in bones and kidneys (100).

Recent studies have shown that stem cell exosomal lncRNAs can combat myocardial aging. For example, Zhu et al. injected hUCMSC-exo-*MALAT1* into the tail veins of aging mice and found that the UCMSC-exos reversed the adverse effects of aging on cardiac function, increased the mRNA expression of the cardiac antiaging marker *TERT*, reduced the mRNA expression of the cardiac aging marker *P21* and the inflammatory factor *TNF-*α, and reduced the protein expression of p-p65 in cardiac tissue, but these antiaging effects were reversed by siMALAT1. Culturing UCMSC-exo-*MALAT1* with senescent cardiomyocytes reduced the activity of *NF-*κ*B* and the expression levels of p-p65 and *TNF-*α, but silencing MALAT1 blocked these effects. These studies suggest that UCMSC-exos can fight cardiac aging through the *MALAT1*/*NF-*κ*B*/*TNF-*α axis (46). Similarly, Xia showed that adipose MSC (ADMSC)-derived exosomes were enriched with *MALAT1* under hypoxic conditions. Hypoxic MSC-exos were cultured with cardiomyocytes and inhibited doxorubicin (Dox)-induced cardiomyocyte senescence. In addition, hypoxic MSCs-exos can improve myocardial cell metabolism disorders. Database and luciferase reporter assays show that hypoxia-induced ADMSCs-exo-*MALAT1* can improve mitochondrial dysfunction in cardiomyocytes after Dox treatment by targeting *MIR-92a-3p*/*ATG4A* and combating cardiac aging (47). In addition, Zhuang et al. found that the lncRNA *NEAT1* was significantly enriched in exosomes derived from BMSCs treated with MIF. MSCs^MIF^-exos can resist Dox-induced myocardial aging in mice and improve heart function. In cell experiments, MSCs^MIF^-exos could also resist Dox-induced senescence in cardiomyocytes by reducing the number of cells in the G0/G1 phase, reducing the expression of the senescence genes *P27* and *P16*, reducing the percentage of SA-β-gal-positive cells, and increasing the telomere length and activity. Database and luciferase reporter assays show that MSCs^MIF^-exos regulate the *MIR*−*221-3p*/*SIRT2* axis by transporting *NEAT1* to protect against Dox-induced cardiomyocyte senescence (52). We agree that stem cells are regarded as a breakthrough point against cell senescence, and the results of exosomal lncRNAs derived from stem cells against cardiomyocyte senescence are encouraging. Furthermore, exosomal lncRNAs provide a new perspective for the treatment of cell senescence and cell metabolic damage.

## Other Cardiovascular Diseases

Wang et al. found exosomal lncRNA crosstalk in chronic kidney disease (CKD) and CVD. The authors showed an increased expression of the lncRNA *ZFAS1* in the hearts of CKD mice, transfected ZFAS1 into human cardiomyocytes (HCMs) and collected exosomes, and found that *ZFAS1* was significantly enriched in HCM-derived exosomes. Injection of HCM-exos into mice significantly reduced their heart function and induced myocardial fibrosis. Similarly, HCM-exos activated the WNT/β-catenin signaling pathway in mouse cardiomyocytes after intervention *in vitro*. Silencing *ZFAS1* can inhibit fibrosis in human CFs (HCFs). Biological database and luciferase assays show that *ZFAS1* is transferred to HCFs through exosomes secreted by HCMs and induces myocardial fibrosis through the *MIR*−*4711-5p*/WNT/β signaling pathway (60). The communication of exosomal lncRNAs between CKD and CVD provides a reference for the study of the relationship between other systemic diseases and CVD.

Luo et al. found 105 significantly upregulated lncRNAs, 126 significantly downregulated lncRNAs, 77 significantly upregulated mRNAs, and 102 significantly downregulated mRNAs in plasma exosomes from five patients with rheumatic mitral stenosis. GO and KEGG analyses of the differentially expressed lncRNA-related genes showed that these cells are involved in magnesium homeostasis, ERBB signaling, RAS signaling, and inflammation. The KEGG pathway analysis of the upregulated mRNAs showed that they are associated with pathways associated with hypertrophic cardiomyopathy and dilated cardiomyopathy. In addition, the analysis of differentially expressed lncRNA subgroups indicated that five pairs of lncRNAs and their accessory-related genes were coexpressed simultaneously; the four downregulated pairs were RP13-820C6.2/*EP400*, RP11-339B21.15/*CERCAM*, G004800/*ZBTB7B*, XLOC_010028/*PDE3A*, and the one downregulated pair was XLOC_004201/*STOX2*. This finding highlights the potential diagnostic and therapeutic role of exosomal lncRNAs in rheumatic heart disease (65).

## Outlook and Summary

Current research concerning exosomes and exosomal lncRNAs is still in its infancy. First, the current methods used for the separation and purification of exosomes are different, and the production capacity is low, which is far from the standard of clinical application. It is of practical significance to upgrade the technology used for the separation and purification of exosomes. For example, Chen et al. optimized the yield and purity of exosomes by using an ultrafast separation system (EXODUS), which was innovated by ultrafiltration (101). We suggest that the separation and purification of exosomes should achieve a high a standard as possible.

Second, the organ targeting of exosomes needs to be examined. Local injection into a target organ may have improved therapeutic effects. However, intravenous injection results in unsatisfactory retention rates of exosomes in the target organ. We agree that the targeting of organs can be increased by surface modification of engineered exosomes, but the low immunogenicity and non-tumorigenicity of exosomes should be retained (102). In addition, heterogeneity is a difficult problem in the study of exosomes (103). The biological and functional mechanisms of serum exosomal lncRNAs from different CVD patients and exosomal lncRNAs secreted by different cells are different. Therefore, we suggest that the separation and purification of exosomes should be carried out under uniform and appropriate standards to the greatest extent possible. Therefore, we should search for traces of exosomes and exosomal lncRNAs in serum or tissues from more CVD patients and perform functional analyses. Furthermore, the exploration of the interaction between cells from different sources and target cells of exosomal lncRNAs should be more groundbreaking. Finally, deciphering the specific molecular mechanism of lncRNA selection and modification in exosomes may introduce new perspectives.

In addition, the relationship between lncRNAs and exosomes is intriguing. In general, exosomes act as carriers of lncRNAs to achieve cell-to-cell transfer and regulation. However, Yang et al. found that increasing the level of the lncRNA *HOTAIR* can promote the secretion of hepatocellular carcinoma exosomes (104). Similarly, Xing et al. found that the lncRNA *HAND2-AS1* can inhibit the level of exo-*MIR-106a-5p* secreted by MSCs (105). In the field of CVD, research by Li showed that the overexpression of the lncRNA *NRON* can significantly inhibit the expression of exo-*MIR23A* secreted by atrial myocytes, promote the polarization of M2 macrophages, and reduce atrial fibrosis (106). The above studies show that lncRNAs, exosomes and lncRNAs and miRNAs in exosomes are closely related and complex. Therefore, we believe that in the CVD field, more attention and action are still needed in the study of the interaction mechanism among exosomes and exosomal lncRNAs and miRNAs.

The application and innovation of engineered exosomes may compensate for the disadvantage of exosomes produced by natural cells. Gene-enriched exosomes can be obtained through physical and chemical approaches, such as electroporation, ultrasound, and liposome-mediated membrane fusion (107). The surface modification of engineered exosomes allows exosomes to target tissues or organs and can be used to monitor the pharmacokinetics of exosomes in the body in real time to produce ideal biological effects (102). Therefore, using the advantages of engineered exosomes can provide reveries for the therapeutic effects of exosomal lncRNAs in the clinical application of real CVD patients. Of course, these applications can only be applied after solving the heterogeneity of exosomal lncRNAs and satisfying their basic conditions as drug carriers. In addition, proteomic and lipidomic studies of exosomes are potential hotspots in the study of CVD. For example, Takov et al. conducted a proteomic analysis of EVs derived from MSCs and found that migration-promoting mediators, such as PTX3, BGN, and RTN4, mediate angiogenesis after myocardial infarction (108). In summary, we believe that exosomes and exosomal lncRNAs may play an important role in the diagnosis and treatment of CVD in the future.

## Author Contributions

ZY wrote the manuscript, figure legends, and created the figures and tables. WH revised the manuscript. All authors contributed to the article and approved the submitted version.

## Conflict of Interest

The authors declare that the research was conducted in the absence of any commercial or financial relationships that could be construed as a potential conflict of interest.
